# Methods of Piloting an Abstraction Tool to Describe Family Engagement in the Hospital Setting: Retrospective Chart Review

**DOI:** 10.2196/66549

**Published:** 2025-06-03

**Authors:** Jennifer Morgan, Jennifer Cahill, Christine Ritchie, Lingling Zhang, Priscilla Gazarian

**Affiliations:** 1 Manning College of Nursing and Health Sciences, University of Massachusetts Boston, Boston, MA, United States; 2 Department of Nursing, Massachusetts General Hospital, 55 Fruit Street, Boston, MA, 02114, United States, 1 6177263336; 3 Yvonne L. Munn Center for Nursing Research, Massachusetts General Hospital, Boston, MA, United States; 4 Division of Palliative Care and Geriatric Medicine, Harvard Medical School, Boston, MA, United States; 5 Mongan Institute Center for Aging and Serious Illness, Massachusetts General Hospital, Boston, MA, United States; 6 Tan Chingfen Graduate School of Nursing, University of Massachusetts Chan Medical School, Worcester, MA, United States

**Keywords:** retrospective chart review, medical record review, patient family centered care, care transitions, family-centered care, medical record, family engagement, patient care, abstraction tool, hospital setting, electronic medical record, EMR, data extraction, decision-making

## Abstract

**Background:**

Family engagement in hospitals is crucial for improving outcomes and ensuring holistic, patient-centered care. However, there is limited understanding of how providers document family engagement in electronic medical records (EMR) and how factors such as race and health disparities influence engagement practices. The absence of standardized EMR templates complicates tracking engagement and assessing its impact on patient outcomes. Retrospective chart review (RCR) is an effective method for investigating clinical practice and how family engagement is documented, using both structured and unstructured data from patient records. Despite its potential, gaps remain in the literature regarding distinctions between the prepilot and pilot phases in RCR studies.

**Objective:**

This article describes the prepiloting and piloting stages in the development of an abstraction tool for an RCR study, highlighting how these phases refined the tool for extracting family engagement data from the EMRs.

**Methods:**

A cohort of 2032 medical records was selected using the Research Patient Database Registry and EMRs. Initially, a draft tool was tested during the prepilot phase to assess its stability. To optimize diversity, the sample was then stratified by race. The modified tool was subsequently piloted on a subset of the sample.

**Results:**

The prepilot phase tested the tool on 9 records. In the pilot phase, the tool was applied to 39 records, representing approximately 10% of the sample. After the prepiloting and piloting phases, 293 of the 405 patient records were deemed eligible for inclusion. More than three-quarters of patients had documentation of presence and communication; whereas, only about one-third had documentation of shared decision-making involving families.

**Conclusions:**

The prepilot phase helped standardize the abstraction tool, align it with the EMRs, and address potential biases. The pilot phase provided insights into data availability and highlighted areas for refinement before finalizing the tool for the remaining records. Together, these phases ensured the tool’s effectiveness for use in large-scale RCR studies.

## Introduction

Family engagement refers to the active involvement of family members in patient care and decision-making processes. This includes their participation in specific aspects or domains of engagement, defined as presence, communication, identification and acknowledgment of family needs, shared decision-making, and contribution to care [1,2]. Family engagement in the care of seriously ill patients is more important than ever in the postpandemic era, especially given concerns about capacity and labor force shortages in acute care settings [3]. Family, as defined by the patient, may be a formal, legal, or informal relationship. Family engagement improves patient outcomes, enhances the patient experience, and ensures that care remains holistic and centered on the patient and their support network [4-7]. Without engagement, hospital stays may be prolonged [8,9], and readmission rates and overall health care utilization tend to increase [10,11]. At the end of a patient’s life, family engagement facilitates healthy bereavement for families and increases job satisfaction among providers [12].

The literature lacks clear insight into how family engagement occurs in hospital settings, which specific domains are experienced, the roles families play during inpatient care, and how these roles are documented in electronic medical records (EMR) [13-17]. Additionally, there is a gap in understanding of how race, ethnicity, and health disparities influence clinicians’ initiation of family engagement [15-17]. The absence of a standardized EMR template further complicates the documentation of family engagement, limiting our understanding of how it affects patient outcomes in both acute care and the transition from acute care [17].

Retrospective chart review (RCR) is an effective methodology for investigating clinical practices, such as the current standard for documenting family engagement in medical records. As a foundational approach in clinical research, RCR involves extracting both structured data (eg, demographic data, age, or length of stay) and unstructured data (eg, nursing notes) from patient records [18-21]. This method provides insights into how clinicians initiate and sustain family engagement, as seen in unstructured data such as progress notes. As RCR research relies on both rigor and replication, careful preplanning, adherence to defined steps, and development of a well-structured abstraction tool is critical [19-23]. The abstraction tool standardizes data collection, reduces bias, and ensures consistency, making replication by other researchers feasible. However, there is a gap in the existing literature in making a distinction between the prepilot and pilot phases of the abstraction tool development. The abstraction tool in this study guided an RCR to assess current documentation standards of family engagement in hospitalized patients at a single academic medical center, while exploring potential variations in documentation based on race or ethnicity, social determinants of health, and patient characteristics, with the study published separately [17]. This paper describes the development of an abstraction tool through two distinct phases—prepiloting and piloting—and how each phase contributed to refining the tool for extracting data on family engagement from the EMRs.

## Methods

### Design, Setting, and Participants

The RCR extracted clinical and sociodemographic data from the EMRs of patients aged 18 years and older who were admitted to one of three neuroscience units at an academic medical center over six months in 2023. These units were selected as their health care teams had received training for serious illness conversations, which encompass communication, presence, needs assessment, and shared decision-making [24]. Exclusion criteria included hospital employees; patients admitted for fewer than three days (as unstructured notes often omit mention of family); those with a history of familial abuse or neglect (due to safety-related restrictions on family engagement); and patients who died during admission, as family engagement at the end of life, while vital for dignity, does not contribute to safe transitions between care settings [12].

### Data Collection

Following institutional review board approval and a waiver of consent, a deidentified and encrypted list of eligible patients was extracted from the Research Patient Database Registry, a data warehouse containing EMR data from hospitals within the academic medical system. The database identified 2032 eligible patients. To optimize a diverse sample, the list was stratified by race into three groups [25]. The final convenience sample (N=405) included 135 Black, 135 White, and 135 Asian or Other patients [26]. Of these, 293 patients met inclusion criteria for data abstraction, exceeding the recommended sample size of 192, which calculated using G*Power (version 3.1.9.6; Heinrich Heine Universität Düsseldorf) with 80% power and a medium effect size to detect differences among the stratified racial groups [27].

A literature review of family engagement revealed significant gaps in existing research. It also helped identify and operationalize key variables of interest (ie,family engagement domains; Table 1). Understanding the structure of the medical record guided the identification of proxy measures for each domain. A draft abstraction tool was developed incorporating both structured and unstructured data using the Research Electronic Data Capture (REDCap, version 14.0.27), a secure web-based data management platform [28]. Nurse and physician notes from admission through two days post-admission, as well as two days prior to discharge through the day of discharge, were reviewed, as these are critical transition points for both patients and families [16,29]. To improve reliability and validity, observations and decisions were noted in an operating manual for both phases of the study [20]. Additionally, using intra-rater reliability, an accepted substitute for inter-rater reliability [22], the abstractor performed two passes through the records, followed by a third pass through any abstraction that had less than 95% agreement [17].

**Table 1.** Table identifying proxy measures of family engagement domains, where they can be found in the supporting literature. The table connects the proxy measures to specific family engagement domains.

Proxy measures	Family engagement domains with definition				
	Presence – Families physically bedside as attendants [1,30]	Family Needs – Family’s distinct needs that must be met to form a partnership [1,30]	Communication – Bi-directional sharing of information between family and provider [1,30]	Shared-decision making – Decision-making related to choices about care [1,30]	Contribution to care – Tangible care (IADLs^a^, ADLs^b^) and intangible care (emotional and moral support) [1,30]
Nursing note [31-33]	✓	✓	✓	✓	✓
Admission MD^c^ note [32]	✓	✓	✓	✓	✓
SW^d^ consult placed [32]		✓			
CM^e^ note [34]	✓	✓	✓	✓	✓
EMR^f^-family education [32,35]	✓	✓	✓		✓
EMR-psychosocial family assessment [33,35]		✓	✓		✓
EMR-family coping [32,33]	✓	✓	✓		✓
EMR-serious illness conversation [33,35]	✓		✓	✓	
Interpreter services [32]		✓	✓	✓	✓
EMR-health care agent [30,32]		✓	✓	✓	
EMR-designated caregiver [30-32]	✓	✓	✓	✓	✓

a IADL: Instrumental activities of daily living.

b ADL: Activities of daily living.

c MD: Medical doctor.

d SW: Social work.

e CM: Case management.

f EMR: Electronic medical record.

### Data Analysis

Data were analyzed using SAS software (version 9.4; SAS Institute). Structured and unstructured data were reviewed for the presence or absence of family engagement domains, rather than their magnitude. Consult notes (eg, physical therapy, occupational therapy, case management, social work, spiritual care, palliative care, hospice) were reviewed if available, particularly focusing on admission and discharge. Descriptive statistics were calculated for continuous variables and counts and percentages for categorical variables [17]. The *χ*^2^ tests were used to assess differences between groups.

### Ethical Considerations

This RCR required institutional review board approval as it involved the analysis of medical records without direct patient interaction and a waiver of consent was granted (Protocol # 2023P003145). The data was deidentified to ensure privacy. Additionally, a patient with an unusually long length of stay was excluded from the study to protect privacy. Confidentiality was maintained throughout the study by securing data behind institutional firewalls, and access to data was limited to study personnel.

## Results

**Figure 1.**
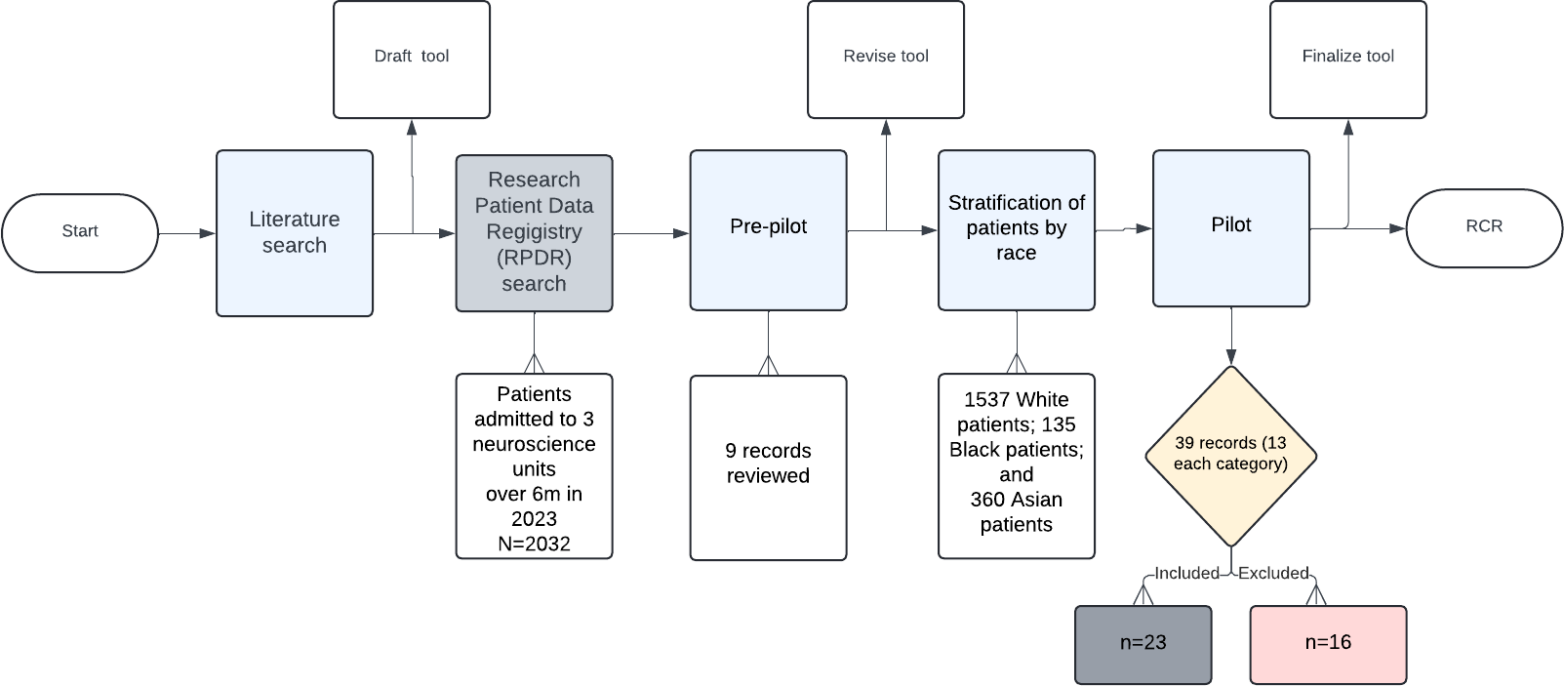
Overview of the early stages of the RCR, illustrating the progression from literature review to pre-pilot and pilot phases, and the interaction with the abstraction tool and sample populations. RCR: retrospective chart review; RPDR: Research Patient Data Registry.

Figure 1 illustrates the flow of the RCR through the prepilot and pilot phase. These two phases clarified the types of data necessary to capture evidence of family engagement, identified where the data were located within the EMR, and showed how family engagement domains were documented. They also highlighted how to address inconsistent data, interpret missing documentation, and finalize the tool for use in the main study. Furthermore, the prepilot and pilot phases uncovered potential threats to the reliability of the tool and offered the opportunity to address them before the larger study.

The RCR included structured and unstructured data from 293 patient records and identified which family engagement domains were captured in the EMR, who documented them, and where they appeared in the EMR [17]. The intra-rater reliability for abstraction ranged from 90% (n=1) to 100% (n=170) [17]. Abstraction revealed that the most frequently documented domains were presence (223/293) and communication (227/293), while the least documented domain was shared-decision making (87/293). Most of the family engagement documentation occurred in unstructured data (eg, nursing notes and physical or occupational therapists). There were no perceived differences in documentation in terms of race, social deprivation, or cognitive impairment [17]. However, there were differences in the domain documentation related to discharge placement and provider discipline [17]. These findings were directly related to the prepilot and pilot phase and the development of the abstraction tool.

### Prepilot Phase

Standard guidelines suggest that the abstraction tool be tested on three to five records to align with the EMR workflow and to address potential reviewer biases and assumptions. During the prepilot, the draft abstraction tool was initially applied to three randomly selected records, where coding errors—such as issues with branching logic—were identified and corrected. Additionally, organizational and formatting changes were made to better align the tool with the EMR structure. The modified tool was then tested on four more records, which identified consult notes that did or did not contain family engagement documentation. Further modifications were made, and the tool remained stable when applied to two additional records. In total, 9 of the aggregate 2032 records were reviewed in the prepilot phase.

### Pilot Phase

For the pilot phase, standard recommendations suggest testing the abstraction tool on 10% of the final sample [28,36,37]. The tool was applied to 39 patient records from the sample population: 13 Black, 13 White, and 13 Asian or Other patients. Six Black and 4 White patients were excluded for being admitted for fewer than 3 days and 6 patients from the Asian or Other group were excluded due to short stays, death during admission, or potential risk of identification due to length of stay. A total of 23 out of 39 patients (59%) were included for pilot phase analysis. There were no significant differences between included and excluded participants when stratified by race. A test of homogeneity indicated that the only distinguishing variable between the groups was the admitting service.

## Discussion

### Primary findings

Using the refined abstraction tool, the RCR identifies which domains of family engagement are documented in the EMR, who records the information, and where it is located [17]. Development of the tool occurs in two distinct but complementary phases—prepiloting and piloting—each playing a critical role in refining the data abstraction process and enhancing the tool’s accuracy and usability.

Prepilot testing involved an initial evaluation to identify issues such as unclear instructions or data elements, with a small group providing feedback for adjustments [22]. Following this, pilot testing with a larger, more diverse sample assessed the tool’s consistency, accuracy, and real-world feasibility, ensuring its readiness for full-scale data collection. These two distinct phases were essential for ensuring the abstraction tool’s reliability and effectiveness in extracting relevant data.

### Prepilot Phase

#### Improve Efficiency

The prepilot phase highlighted opportunities to streamline the abstraction process. Initially, the tool lacked an intake form to assess eligibility and additional items to guide the abstraction of proxy measures. The first revision integrated the intake form using branching logic to capture all reviewed records in one form while being able to distinguish patients who met the inclusion criteria from those who ultimately did not. Additionally, the order of the items appearing within the tool was changed to more closely reflect the EMR workflow and maximize efficiency (eg, age was moved to the first item as it appears before other demographic items). Further modifications addressed gaps in instances where engagement domains were documented and by whom, including the omission of physical and occupational therapist notes, which consistently identified the patient’s social support. A search term was identified to improve abstraction efficiency.

#### Identify Assumptions

The prepilot phase also challenged several assumptions in the draft tool. For example, it was initially assumed that the emergency room physician and nurse notes would be the primary sources of family engagement data. However, admitting service history and physical examination notes as well as the nurses’ admission notes were found to provide more relevant documentation of family engagement.

### Pilot Phase

#### Identify Potential Problems

The pilot phase raised concerns about excluding patients with stays shorter than 3 days. This exclusion criterion removed more patients than anticipated, particularly those admitted for 2 days. A posteriori, this criterion was re-evaluated. Allowing 2-day stays would have increased inclusion from 23 of 39 patients (59%) to 31 of 39 patients (79%). However, the rationale for excluding these patients—namely, the possibility that abbreviated admissions may not allow insufficient time to initiate family engagement in the hospital—remained compelling, and the exclusion criterion was retained. Additionally, “extended admission” was added as a new exclusion criterion due to confidentiality concerns.

#### Identify Missing Variables or Indicators

The pilot phase also identified missing variables that could inform data analysis and interpretation. For example, initially, the tool relied upon cognitive impairment being added to the patient’s list of problems. As cognitive impairment is underdiagnosed and under-recognized in acute care settings, [38], broader criteria were adopted to identify patients who were experiencing cognitive impairment. The Confusion Assessment Method (CAM), a standardized tool for assessing delirium was added as a proxy measure of cognitive impairment, along with the use of direct observers and restraints. Being identified as cognitively impaired, “delirious” or Confusion Assessment Method-positive could influence family engagement, as these patients would likely need additional support from their families and be unable to initiate engagement themselves, and therefore need providers to proactively engage with families. The tool was revised to include whether a patient was delirious and whether sitters or restraints were used for safety. Additionally, documentation of family meetings for critical patients in one of the neuroscience units was included in the tool.

#### Identify Data That Need Clarification

Finally, the pilot study uncovered items that required clarification [20,28]. One purpose of the pilot was to ensure consistency of abstraction and coding [37]. In unstructured data such as nursing narrative notes, “supportive” was used to describe some family members but not others. Upon reflection and team consensus, terminologies such as “supportive family bedside” were considered documentation evidence for two domains, including presence and contributing to care. Similarly, some providers documented the existence of family support even when the family was not physically present. However, modification to the tool was made to acknowledge social support, allowing for the possibility that the support was not physically present at the patient’s bedside.

#### Preplanning

Conducting prepilot and pilot phases allowed for preplanning. The two distinct phases helped refine the study timeline [39]. It was found that abstracting each record in the pilot phase took approximately 15 to 20 minutes. Knowing this enabled the team to estimate the data collection timeframe based on the known sample size and allocate resources accordingly.

### Limitations and Strengths

Key strengths of RCR methodology include its flexibility, cost-effectiveness, ability to access unique information, reduction of recall bias, and potential for creating randomizable sample frames [19]. Beyond epidemiological and clinical research, RCR is useful for evaluating care patterns and informing training [19,20,28]. However, a notable limitation was that for an RCR, data are obtained twice from the patient, family, or clinical scenarios; first, the clinician gathers the data and second, they document the data. Finally, while every effort was made to optimize sample diversity, stratifying by race—a social construct—can introduce bias if patients did not self-identify their race. These characteristics should be kept in mind during the prepilot and pilot phases of the abstraction tool development.

### Conclusion

The prepilot and pilot phases were essential for refining the abstraction tool used in this RCR. These phases allowed the tool to be optimized for efficiency and clarity, addressing potential problems, and improving data collection. The prepilot phase involved an initial evaluation to identify issues such as unclear instructions or missing data elements, with a small group providing feedback for adjustments. On the contrary, the pilot phase involved a more extensive and structured evaluation, with a larger sample size and more diverse set of users. The final abstraction tool integrated both structured and unstructured data, capturing family engagement across multiple disciplines, including nursing, case management, physicians, and ancillary consultants. The focus remained on critical transition points in care (ie, admission and discharge). The two distinct phases ensured that the tool was clear, efficient, and comprehensive, setting the stage for the larger study.

This study addresses a previously unexamined aspect of RCR. Describing the distinction between the prepilot and pilot phases increases transparency and increases the rigor and reproducibility of the methodology. The description of the iterative testing within each of the two phases, indicating that it was only adopted after careful systematic evaluation, also highlighted the learnings and rationale underlying specific design decisions. This step-wise approach to RCR helps to build best practice standards and inform policy while documenting family engagement.

## Abbreviations

EMR: electronic medical record
RCR: retrospective chart review
